# Hippocampal relational memory and spatial reconstruction: where are we at and where are we bound?

**DOI:** 10.3389/fcogn.2026.1876456

**Published:** 2026-08-11

**Authors:** Taylor K. Brown, Ioannis Valoumas, Michael R. Dulas

**Affiliations:** Department of Psychology, Binghamton University, Binghamton, NY, United States

**Keywords:** hippocampus, reconstruction, relational memory, spatial memory, spatial reconstruction tasks

## Abstract

Memory is increasingly understood as a reconstructive process, yet many memory paradigms assess only the outcome of retrieval rather than the structure of reconstruction itself. Spatial reconstruction tasks (SRTs) provide a powerful approach for examining reconstructive memory because they require participants to recreate studied object-location configurations and allow researchers to quantify multiple sources of spatial error. This review synthesizes theoretical and empirical work on SRTs as measures of hippocampally-mediated relational memory. Lesion studies established that hippocampal damage impairs spatial reconstruction as well as demonstrating that SRTs are sensitive to particular underlying mechanisms. SRTs have been utilized across amnesia, traumatic brain injury, aging, and development, with accumulating evidence suggesting that they index the role of the hippocampus in binding specific objects to specific locations. However, the literature is limited by substantial variability in task design, stimulus type, and analytic metrics. We review commonly used measures and differing interpretations across studies using SRTs. Finally, we outline recommendations for standardizing SRT design and analysis, discuss key limitations and methodological constraints that should be addressed, and highlight future directions involving eye-tracking, neuroimaging, clinical populations, and extensions beyond spatial memory. Overall, we argue that SRTs offer a sensitive framework for understanding how relational memories are encoded, bound, and reconstructed, and have great utility for future research avenues.

## 1 Introduction

Memory is not like taking a video that can simply be replayed later; instead, memory is increasingly understood as a reconstructive process. At encoding, we build a representation of an event based on how we interpret and organize incoming information. At retrieval, we rebuild that representation, drawing on both stored traces and current cognitive processes. Neither of these stages acts in isolation: the way you encode is shaped by the retrieval of previous related information, and retrieval involves reconstructing and re-encoding information in ways that can alter its organization. This framework emphasizes that reconstruction shapes both the initial formation of a memory and its subsequent retrieval. Yet, while memory is reconstructive in nature, most memory studies do not assess reconstruction directly, often sampling only the final “product” of retrieval. Although reconstruction cannot easily be assessed in all memory domains, the field of spatial memory lends itself to be evaluated under the lens of reconstruction. Spatial memory can be tested using spatial reconstruction tasks (SRTs), which are particularly powerful as they provide sensitive measures of both memory accuracy and precision. In SRTs, participants study a configuration of multiple items in space, and after a delay, they attempt to recreate the configuration, by placing the objects back to their original position. This procedure allows researchers to assess not only memory for the remembered locations, but also the relational structure among items in the array. SRTs provide rich data that can be used to quantify overall spatial error and identify underlying sources of misplacement, including relational binding errors, location memory precision, and item-item spatial relationships. Spatial reconstruction metrics can also dissociate relational memory performance across multiple groups, including in amnesia, Traumatic Brain Injury (TBI), aging, and development. Further, combining SRTs and eye-tracking can provide insights into hippocampally-mediated information sampling and strategic viewing behavior. Moreover, studies coupling SRTs with neuroimaging data have clarified the key role of the hippocampus in spatial reconstruction.

However, substantial variability exists in how spatial reconstruction tasks are designed and how their data are analyzed, resulting in inconsistent indices of spatial memory across studies. Additionally, while a great deal of focus has been placed on the role of the hippocampus in spatial reconstruction, less is known about the role of other regions in such tasks. The goal of this paper is to evaluate the varied approaches to spatial reconstruction, in terms of both paradigms and analyses, in order to establish best practices for analyzing and assessing spatial reconstruction data. Secondly, and perhaps more importantly, this paper looks to synthesize what spatial reconstruction research has uncovered about hippocampal relational memory, what gaps remain in the literature, and point to future avenues of research questions, both within the domain of spatial memory and beyond. The present paper first reviews theoretical and empirical work on spatial relational memory, then examines variability in SRTs, first focusing on their associated metrics, as well as their paradigm implementation, and finally outlines recommendations for SRT use going forward and its implications for understanding spatial and relational memory processes.

While spatial memory encompasses a broad range of metrics and underlying mechanisms, this review focuses on studies featuring spatial reconstruction, anchored in the designs first used in hippocampal lesion patients (Smith and Milner, 1981, 1984). To that end, our review categorizes studies as being “spatial reconstruction” if they meet the following criteria:

1. **Free reconstruction of spatial locations:** Studies must have allowed for and assessed the ability to freely place items in space during retrieval, in any order and without time constraints. Thus, testing a spatial “context” (e.g., left vs. right, top vs. bottom) or a small grid of locations would not qualify.
2. **Multiple items in the studied arrays:** Studies must have required participants to remember the locations of multiple items in an array.
3. **Multidimensional assessment:** Studies were included if they assessed spatial reconstruction using multiple outcome metrics rather than a single global accuracy score. Thus, studies with binary “correct/incorrect” spatial memory assessments were not included.

That said, we will frame SRTs in the broader spatial memory literature, both in the background and in the discussion and future directions. Further, while we will focus on what can be considered “allocentric” spatial reconstruction tasks, we will also discuss parallels between spatial reconstruction and spatial navigation.

## 2 Spatial (relational) memory: a brief background

The hippocampus has long been recognized as critical for spatial memory, particularly with the identification of place cells, neurons in the hippocampus which fire selectively when an individual occupies a specific location within an environment (O'Keefe, 1976). More recent research has shown that place cells function as part of a broader network of cells in the Medial Temporal Lobe (MTL). This cell network in the MTL is composed of different cell types, including grid, border, and head direction cells, all specialized for processing space (Hafting et al., 2005; Moser et al., 2008). Within this spatial network, place cells code specific locations, and grid and head direction cells contribute to directional information, collectively contributing to a coherent and flexible representation of space (Moser et al., 2008). The discovery of place cells led to “cognitive map” theory, which proposes that the hippocampus constructs internal, map-like representations that guide navigation and support flexible spatial memory (O'Keefe and Nadel, 1978). Cognitive map theory further suggests that this internal map functions as a spatial framework, represented as a Euclidean coordinate system, which supports the organization of environmental information. Cognitive map theory has been supported with evidence from both behavioral and neuroimaging studies (Ekstrom et al., 2003; Farzanfar et al., 2023). Findings indicated that both the hippocampus and entorhinal cortex form map-like spatial codes, while other regions support the anchoring of maps to landmarks within an environment (Ekstrom et al., 2003; Epstein et al., 2017). This dissociation between hippocampus and MTL has been further strengthened via intracranial recordings, which have demonstrated that hippocampal neurons preferentially fire in response to specific spatial locations, whereas parahippocampal neurons are more responsive to views of salient landmarks (Ekstrom et al., 2003). These findings suggest the MTL has multiple interactive mechanisms that support spatial processing, both in service of spatial memory and navigation.

However, the hippocampus and MTL have also been tied to episodic memory, most famously via patient HM (Milner et al., 1968). Further, there is evidence that place cells can also code for temporal information, suggesting the hippocampus processes other types of relations besides space (Eichenbaum, 2014). Thus, the role of the hippocampus in both spatial and episodic memory has led to an important question as to whether hippocampal representations are specific to space or reflect a more general mechanism for organizing relationships among elements of an experience (Eichenbaum, 2017a). Relational memory theory suggests that the hippocampus supports the flexible binding of arbitrary relations among items, regardless of whether those relations are spatial in nature (Eichenbaum and Cohen, 2001). It has been proposed that cognitive map theory and relational memory theory can be reconciled by viewing cognitive maps as part of a broader relational memory system (Eichenbaum and Cohen, 2014; Ekstrom and Ranganath, 2018), though this view is not always agreed upon (Cohen and Eichenbaum, 1991). Within relational memory theory, the hippocampus supports the flexible binding of relationships among elements of experience, such as items, locations, and contexts, allowing these relationships to be organized within structured representational spaces. Indeed Konkel et al. (2008) demonstrated that individuals with hippocampal lesions show impairments in relational memory across multiple domains, not limited to spatial information. These findings suggest that spatial memory deficits associated with hippocampal damage may reflect broader disruptions in relational binding processes. Consistent with this perspective, the involvement of the hippocampus in both spatial and episodic memory suggests that its function is not restricted to a single domain (i.e., spatial or episodic), but instead reflects a more domain-general involvement in supporting the binding of relational information across contexts. While this may seem tangential to the question at hand, we argue that understanding the relational aspect of spatial memory is a key feature of the evolving field of spatial reconstruction.

Importantly, although spatial and relational memory both rely on the hippocampus, other regions are critical for their success. As previously noted, the surrounding MTL plays a key role in spatial memory, and is also critical relational memory (Ploner, 2000; Aminoff et al., 2007; Mullally and Maguire, 2011). Further, the prefrontal cortex (PFC) contributes to the strategic encoding, organization, and retrieval of representations in a goal-directed manner (Pandya and Barnes, 1987; Preston and Eichenbaum, 2013). This has often been framed through the metaphor of a railroad track, where the hippocampus “lays the tracks” while the PFC allows for switching between those tracks (Eichenbaum, 2017b). Although this metaphor is useful in explaining some aspects of hippocampal-PFC interactions in memory, it may not fully capture memory's reconstructive nature. More specifically, we argue that the “tracks” are not fixed pathways, but are continuously reorganized as memories are retrieved, updated, and re-encoded. In this view, the PFC's role must include guiding attention toward task-relevant relationships during encoding, supporting the controlled construction of relational representations, and facilitating the subsequent reconstruction of these relationships during retrieval. Therefore, the hippocampus and PFC must interact with one another at multiple stages to support spatial memory. Indeed animal models provide evidence of coordinated activity between the hippocampus and medial PFC (mPFC) in support of spatial encoding, retrieval, and spatial working memory (Churchwell et al., 2010; Churchwell and Kesner, 2011). The many subregions and roles of the PFC involved in spatial and relational memory are beyond the scope of this paper, but they include 1) Ventromedial PFC, which supports the integration of information both within and across episodes (Preston and Eichenbaum, 2013; Hebscher et al., 2016), 2) Ventrolateral PFC, which supports controlled encoding, maintenance, and retrieval processing (Postle et al., 2000; Blumenfeld and Ranganath, 2007), and 3) Dorsolateral and anterior PFC, which enable manipulation of relational elements and post-retrieval monitoring (Henson et al., 1999; Hayes et al., 2004; Blumenfeld et al., 2011), among others. Consequently, successful reconstruction of relational memory, including spatial relations, likely requires hippocampal-prefrontal coordination and interaction.

The parietal cortex is also well known to be critical for spatial processing, even in perceptual tasks (Heilman et al., 2000; Husain and Nachev, 2007). Consistent with its role in spatial processing, the parietal cortex has also been implicated in both spatial (Save and Poucet, 2009; Marchette et al., 2014) and episodic memory (Wagner et al., 2005; Cabeza et al., 2008; Gilmore et al., 2015), likely via its connection with the hippocampus (Cabeza et al., 2008; Sestieri et al., 2017) and PFC (Benoit and Schacter, 2015; Robin et al., 2015). Taken together, neural data suggest spatial memory involves a complex set of regions and processes centered around hippocampal relational memory. To date, few studies have directly assessed the role of these regions in reconstruction performance (see Cooper and Ritchey, 2019 for one such example). Thus, as we continue, the caveat should be made that SRTs have focused almost exclusively on the role of the hippocampus, stemming in part from their beginnings in hippocampal lesion studies. However, as we will outline, parallels may exist with other spatial research fields (e.g., navigation), but more work is needed to disentangle the contributions of other regions to spatial reconstruction performance.

### 2.1 The impact of hippocampal lesions on spatial reconstruction

**Figure 1:**
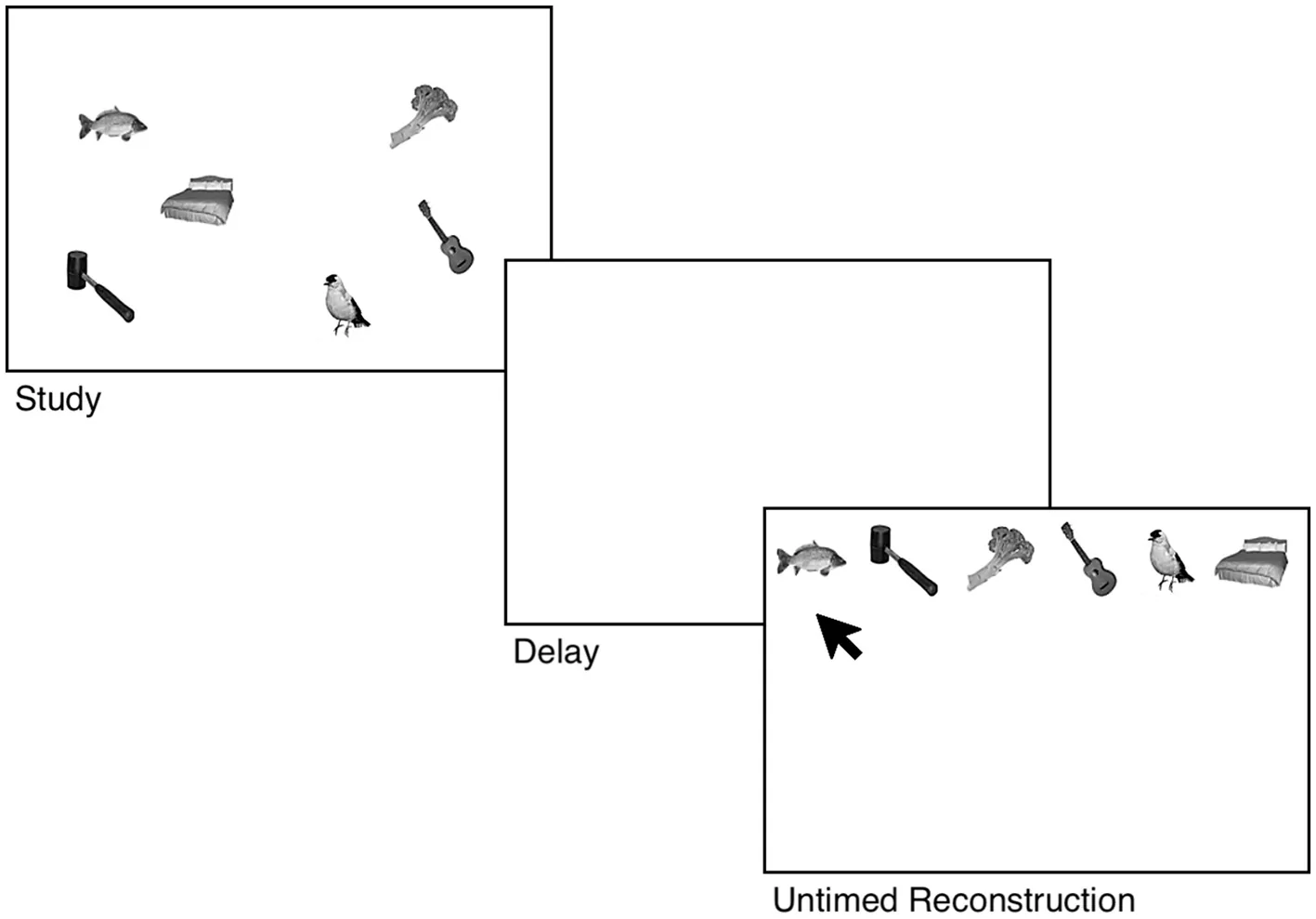
Example trial sequence for a spatial reconstruction task. Depicts computerized SRTs with real-world objects.

Spatial Reconstruction Tasks emerged as a tool for assessing hippocampally-mediated spatial memory in the 1980′s but have evolved considerably over time. However, all SRTs share two key features: 1) allowing participants to manipulate stimuli and reconstruct their locations, and 2) measuring how closely participants reconstructed stimuli relative to their studied locations. An example of a relatively standard, computer-based SRT is shown in Figure 1. In this example six real-world objects are presented during study, followed by a brief delay during which a blank screen is presented to participants. This is followed by an open-ended reconstruction period where the items that were presented during study are placed at the top of the screen and participants can click and drag the items to their studied locations using the computer mouse.

While the earliest SRTs typically presented participants with an array of real objects placed on a tabletop or board (Smith and Milner, 1981), the objective of the task is still comparable to more recent adaptations. Participants studied these objects, often with an incidental encoding task (e.g., attend to their prices), and then attempted to reconstruct the display by placing them back into their original locations. In their initial work, Smith and Milner (1981) found that bilateral, as well as right lateralized hippocampal damage particularly impaired spatial memory, including memory for both the exact positions of the objects as well as the relative locations between objects. These spatial memory deficits were seen even with immediate test and dissociated from item recall deficits. Further research revealed that patients with unilateral right prefrontal lesions displayed intact spatial location memory, despite deficits in the price estimation task, confirming that impaired spatial memory may stem from specifically from hippocampal damage (Smith and Milner, 1984). Later work demonstrated that the extent of damage to the right hippocampus was a strong predictor of spatial memory deficits (Crane and Milner, 2005). These early studies made one thing abundantly clear: the hippocampus, particularly the right, is critical for spatial reconstruction performance, even at short delays.

From these early SRT studies, researchers began shifting the central question from *whether* hippocampal damage affects spatial reconstruction to *how* hippocampal damage alters spatial reconstruction performance. Subsequent studies in patients with bilateral hippocampal lesions aimed at clarifying the nature of hippocampally-mediated deficits in spatial memory, investigating whether deficits were tied to processes such as forgetting (Smith and Milner, 1981), working vs. long-term memory demands (Jeneson et al., 2010), or relational binding (Watson et al., 2013; Horecka et al., 2018). (Jeneson et al. 2010), demonstrated that the hippocampus is particularly critical when relational memory demands exceed working memory capacity (3–4 items), with impairments emerging abruptly beyond at larger set sizes. These findings suggested that deficits in relational memory are tied to impairments in long-term memory. In contrast, Watson et al. (2013) showed that spatial memory deficits are not simply due to reduced precision, but are primarily driven by relational binding errors. Specifically, their data suggested that swaps, where the relative position of items are flipped, are the predominant type of error in hippocampal lesion patients. Critically, patients with hippocampal lesions demonstrated increased swap errors even at short delays and with set sizes as small as two items, well within the domain of working memory. The more recent, computer-based environments, have allowed for more experimental control. Horecka et al. (2018) employed a computerized SRT with hippocampal lesion patients, aiming to develop more precise characterization of their spatial memory deficits. Their data clarified that spatial memory impairments are driven by deficits in the binding of objects to locations rather than a generalized spatial memory deficit. Together, these studies make it clear that the hippocampus is critical for spatial memory, with at least some studies suggesting it plays a critical role in binding objects to locations. However, there is still some debate as to whether the hippocampus is *necessary* within the realm of working memory, as data has been equivocal on this point. Some of this debate may stem from differences in how spatial memory performance is assessed across studies. Indeed, while all SRTs have included a measure of Euclidean distance error (displacement or misplacement), each task included unique measures of relational memory. As the field has continued to evolve and move away from hippocampal lesion work, these inconsistencies in SRT measurement have become further compounded. Thus, it is critical to discuss the measurements that have been developed, and the pros and cons of each, before surveying the more recent literature and the distinctions between various tasks.

## 3 The many measures of SRT

A key advantage of SRTs is that they are relatively simple to conduct, yet provide rich data about spatial memory abilities. However, as noted, the way these data are subsequently handled across studies differs substantially, potentially underlying the inconsistencies in their interpretation. Here we will discuss three classes of measures: 1) **Misplacement** measures, which assess the distance of a reconstructed item from its studied location, 2) **Swap** errors, which attempt to assess pairwise switches between two studied items' locations, and 3) **Assignment Accuracy**, which seeks to directly assess how well participants bound objects to their individual locations, while also allowing for an assessment of overall location memory. These, along with other less common measures, and their history of use are summarized in Table 1.

**Table 1:** Commonly used metrics in spatial reconstruction tasks across studies.

Group	Metric	Measurement	Process	Articles using this metric
Misplacement errors^a^	Misplacement/Raw Misplacement	Average distance between each studied item location and its reconstructed location	Overall reconstruction accuracy/precision	Watson et al., 2013; Horecka et al., 2018; Clark et al., 2017; Rigon et al., 2020; Schwarb et al., 2016; Smith and Milner, 1981, 1984; Crane and Milner, 2005; Tinney et al., 2023; Hassevoort et al., 2017, 2018, 2020
	Remapped Misplacement	Spatial error after removing item identity through optimal matching	Memory for locations independent of item identity	Horecka et al., 2018; Rigon et al., 2020; Cannavale et al., 2019; Hassevoort et al., 2017, 2018, 2020
	Post-Transformed Misplacement	Residual spatial error after removing identity errors and global transformations	Item-specific spatial precision after accounting for global distortion	Horecka et al., 2018; Rigon et al., 2020; Cannavale et al., 2019; Hassevoort et al., 2017, 2018, 2020
Swap errors	Swap (axis) errors	The relative position on each x and y axis for a pair of items is flipped	Identity-location binding failure; compound relational error	Watson et al., 2013; Lucas et al., 2019, 2023; Monti et al., 2015
	“True” swaps and cycle errors	Either two (swap) or more (cycle) items are misassigned to each other's studied locations in a closed loop	Compound relational binding error	Horecka et al., 2018; Rigon et al., 2020; Cannavale et al., 2019; Hassevoort et al., 2017, 2018, 2020
Assignment accuracy	Accurate Assignments	Items placed in their own studied locations	Identity-location binding	Horecka et al., 2018; Rigon et al., 2020; Cannavale et al., 2019; Tinney et al., 2023; Hassevoort et al., 2017, 2018, 2020; Johnson et al., 2018
	Misassignments	Items placed in another item's studied location	Identity-location binding failure	Horecka et al., 2018; Rigon et al., 2020; Cannavale et al., 2019; Schwarb et al., 2024; Hassevoort et al., 2017, 2018, 2020
Global errors	Translation	Uniform shift of the entire reconstructed array	Global spatial displacement bias	Horecka et al., 2018; Rigon et al., 2020; Cannavale et al., 2019; Hassevoort et al., 2017, 2018, 2020
	Scaling	Compression or expansion of the reconstructed array	Global distortion in remembered array size	Horecka et al., 2018; Rigon et al., 2020; Cannavale et al., 2019; Hassevoort et al., 2017, 2018, 2020
	Rotation	Rotation of the reconstructed array around a central point	Global orientation error	Horecka et al., 2018; Rigon et al., 2020; Cannavale et al., 2019; Hassevoort et al., 2017, 2018, 2020
Item-item errors	Relative location accuracy	Whether objects are placed correctly relative to one another	Item-item relational memory	Smith and Milner, 1981, 1984; Jeneson et al., 2010
	Edge resizing	Change in the length of vectors between items	Item-item relational memory	Watson et al., 2013
	Edge deflection	Change in the direction/angle of vectors between items	Item-item relational memory	Watson et al., 2013
	Rearrangement	Degree to which the overall shape/configuration of the array is preserved	Global configuration	Watson et al., 2013
Composite metrics	Composite distance errors (SRd)	A composite metric consisting of: Raw Misplacement and Edge Resizing	Overall reconstruction accuracy/precision and Item-item relational memory	Clark et al., 2017; Schwarb et al., 2016; Daugherty et al., 2020; Delgorio et al., 2022
	Composite arrangement errors (SRa)	A composite metric consisting of: Rearrangements and Swaps	Global configuration and compound relational error	Clark et al., 2017; Schwarb et al., 2016; Daugherty et al., 2020; Delgorio et al., 2022

Metrics are grouped according to the primary spatial or relational process they are thought to assess. ^a^Since some form of misplacement or distance-based reconstruction error is reported in nearly all spatial reconstruction task studies, the articles listed under misplacement errors are intended as representative examples rather than an exhaustive list. Terminology and scoring procedures may vary across studies.

### 3.1 Misplacement

**Figure 2:**
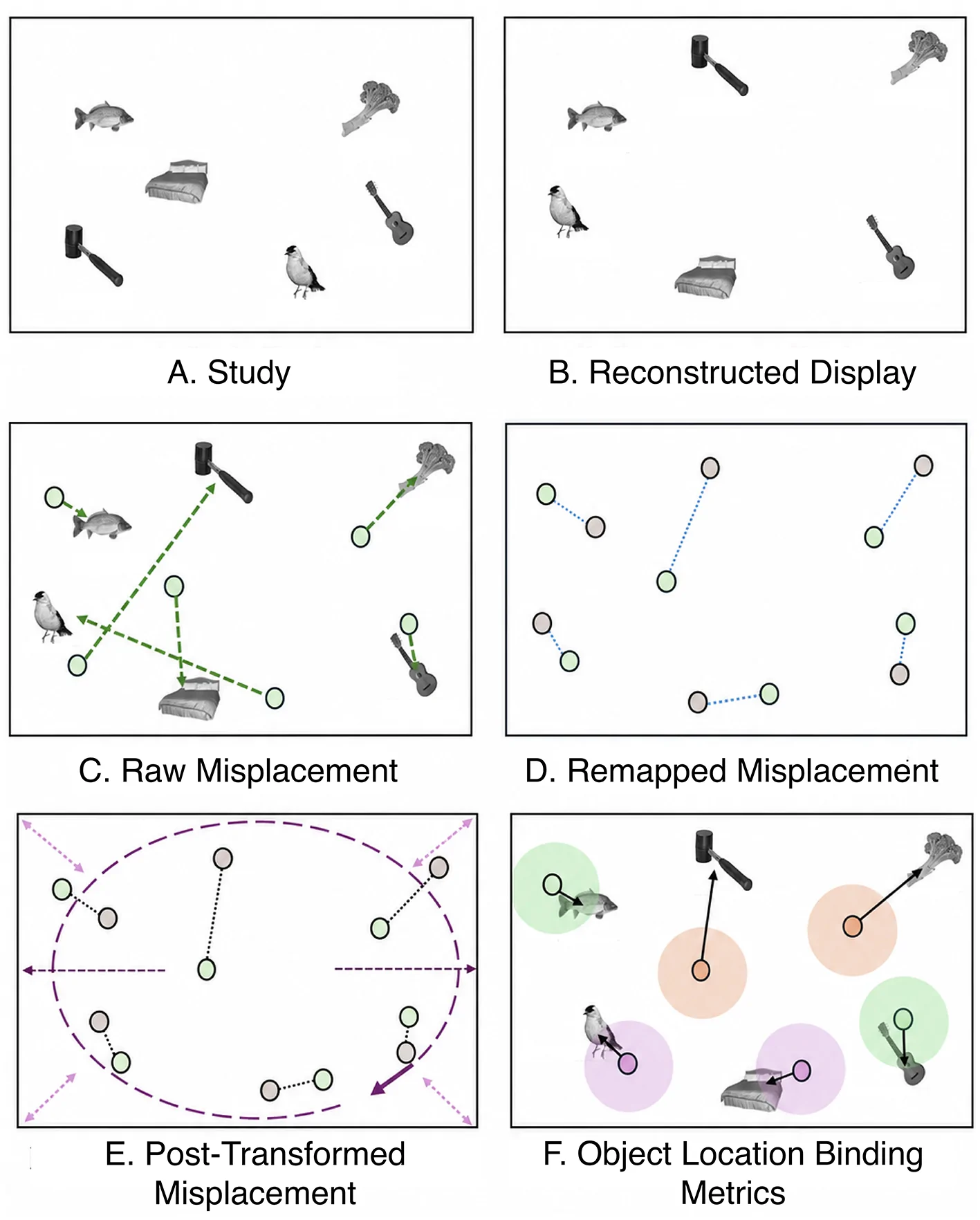
Illustration of spatial reconstruction misplacement error metrics and assignment accuracy in a spatial reconstruction task. **(A, B)** show an example studied display and reconstructed display, respectively. **(C–F)** illustrate four reconstruction metrics. In **(C)**, green dashed arrows show Raw Misplacement, or the distance between each studied item location and its reconstructed location. In **(D)**, blue dotted lines show Remapped Misplacement, or error after item identities are reassigned to minimize total spatial error. In **(E)**, black dotted lines show Post-Transformed Misplacement, or residual error after removing global translation, rotation, and scaling, illustrated by the purple arrows and dashed ellipse. In **(F)**, black arrows show object-location assignments, and shaded circles indicate whether each reconstructed item was assigned to its correct studied location. Green circles indicate Accurate Assignments, pink circles Misassignments (a studied location but the incorrect item), and orange circles indicate location accuracy errors (a studied location that was not used in reconstruction).

Misplacement, or sometimes referred to as displacement, is a direct measure of the distance each object is reconstructed relative to its studied location, be it in physical distance (e.g., Smith and Milner, 1981) or in pixel space (Horecka et al., 2018). In displacement, error is measured by determining how far an object was placed from its true original position, capturing not just the accuracy of item placement, but also the precision of spatial memory. Most, if not all SRT studies that have employed an SRT have included such a measure as a raw assessment of overall performance. However, as discussed, misplacement error can stem from many underlying causes, be it swaps, object-location binding errors, or global transformations. Horecka et al. (2018) introduced a processing pipeline that sought to further interrogate the nature of misplacement during spatial reconstruction of real-world objects on a computer (Figures 2A, B). As with other studies, the authors first calculated **Raw Misplacement** (Figure 2C). Then they anonymized the items and remapped the anonymized reconstructed points to study locations in a manner that globally minimized reconstruction error, leaving a new metric: **Remapped Misplacement** (Figure 2D). Remapped Misplacement can be thought of as the overall distance error of the entire array from its studied configuration, ignoring individual object-location binding demands. Next, they applied a global transformation to the reconstructed array (Figure 2E) to account for global shifts in the reconstructed array, including x, y translations, array rotations, and scaling changes (shrinking or enlarging). This global transformation step produces both a **Post-Transformed Misplacement** measure (Figure 2F), as well as outputs on the magnitude of each of those global error types^1^.

Combining these three levels of Misplacement assessment, Horecka et al. (2018) uncovered an important set of findings. First, as with previous studies, bilateral hippocampal lesions resulted in greater Raw Misplacement relative to matched control participants. However, the remapping step massively reduced these group differences, though patients with hippocampal lesions still showed significantly more Remapped Misplacement. Lastly, while global transformation correction did further reduce overall misplacement error, it did so equally for patients and controls. Still, even after these steps, patients with hippocampal lesions showed significantly more misplacement than controls. Based on the large decrease in misplacement error in the lesion patients after remapping, the authors suggested that hippocampal damage is particularly tied to binding identities of items to their specific locations, rather than a general tendency to globally shift the array. Although this work, along with other studies, largely focused on investigating object-location binding deficits, the remaining deficits in Post-Transformed Misplacement are worth considering for a moment. The remaining misplacement has often been referred to as “local” misplacement, i.e., item-specific rather than global. However, the nature of this “local” misplacement remains unclear, particularly given the prevalence of significant group differences in this final misplacement measure. Specifically, it is unknown whether Post-Transformed Misplacement represents a metric of overall precision in hippocampally-mediated spatial memory (Ekstrom and Yonelinas, 2020), or whether it reflects the propensity for large scale errors (e.g., reconstructing an item in an entirely unstudied location) that are not corrected by remapping or global transformation. Likely, it reflects a combination of both, but the balance between those possibilities, and how that balance shifts across various populations may warrant further investigation. However, like the field itself, we now shift our focus to the nature of those relational errors, and the claim that they reflect object-location binding effects.

### 3.2 Spatial relational errors—a choose your own adventure?

While SRTs have been relatively consistent in their application of misplacement, there has been little-to-no standardization of follow-up assessments of relational errors. As outlined in Table 1, potential relational errors have been indexed as: 1) **Relative Location Accuracy**, assessing whether objects were placed correctly relative to one another (Smith and Milner, 1981, 1984; Jeneson et al., 2010), 2) Swaps, or what we will refer to as **Axis Swaps**, assessing whether the x, y vector connecting each pair of objects was reversed between study and test (introduced in Watson et al., 2013), and 3) **Assignments** (and Misassignments) (Figure 2F), assessing whether objects were placed in their studied location (within a standard error window), another studied item's location, or in a completely non-studied location (introduced in Horecka et al., 2018). Each of these measures has been used in multiple other places, and sometimes renamed or recombined (see Table 1). Here, we will explore whether the inconsistency in findings stems not from inconsistent data, but from inconsistent analyses.

**Figure 3:**
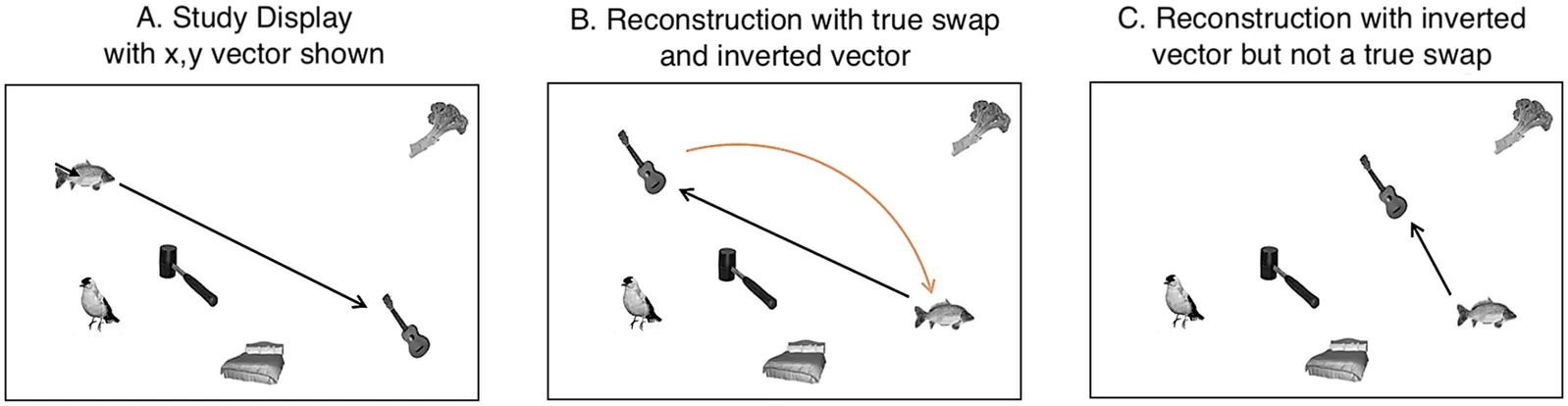
Illustration of true swaps and inverted vectors in spatial reconstruction. **(A)** shows a studied display with the x, y vector between two items. **(B)** shows a reconstruction in which the two items have been placed in each other's studied locations, producing both an inverted vector and a true swap. The orange curved arrow highlights the swapped item locations. **(C)** shows a reconstruction in which the vector between the two items is inverted, but the items are not placed in each other's original locations; therefore, the error reflects an inverted vector but not a true swap.

First, it is important to acknowledge that studies using SRTs with hippocampal patients have consistently reported some form of spatial relational deficit, suggesting that the evidence is clear in this respect. However, the exact nature and time domain of these deficits remains less clear. Much of the field has embraced Swap errors based on both compelling data and a compelling interpretation (Watson et al., 2013; Monti et al., 2015). The study conducted by Watson et al. (2013) concluded that Axis Swap errors were the dominant error type in hippocampal lesion patients, relative to other types of relational errors. The findings revealed that patients frequently made Axis Swap errors, even for spatial arrays containing as few as two items, and even after very short delays, whereas comparison participants rarely made such errors. The conclusion was that, at least within the confines of SRTs, the hippocampus supports the binding of objects to locations, and that damage to the hippocampus led to the confusion of items' locations in the array. The utility of Axis Swap errors as a measure of hippocampal function was later solidified in studies with aging (Clark et al., 2017), eye-tracking (Lucas et al., 2019, 2023), and neuroimaging (Schwarb et al., 2016; Daugherty et al., 2020; Delgorio et al., 2022), suggesting this measure effectively captures both hippocampal function and dysfunction. However, despite its rising use, questions remained about the true nature of Axis Swap errors. As outlined, Axis Swaps represent an inversion of the x, y vector between two reconstructed items relative to their studied locations (Figure 3A). While the implication is that such an inversion means the items switched locations (Figure 3B), multiple other reconstruction outcomes could produce a “Swap.” For example, an Axis “Swap” could be produced even if both items are in entirely incorrect locations (Figure 3C) or even when one item is in its correct location but the other is reconstructed on the opposite side. Moreover, making a large-scale error on a single item, such as taking an item studied in the upper left corner and reconstructing it in the bottom right corner, would produce multiple “Swap” errors, even if all other items had been reconstructed relatively accurately. In short, despite their somewhat pervasive use, the actual underlying cause of Axis Swaps remains unclear.

In an attempt to rectify this uncertainty in Axis Swaps, Horecka et al. (2018) instead aimed to assess accuracy, rather than error. Building on their Misplacement analysis pipeline, they developed measures that we will refer to as Accurate Assignments^2^ (placing a studied item in its studied location), Misassignments (placing a studied item in another studied item's location), and general Location Memory (placing items in studied locations). These measures were assessed after accounting for global transformation error and consisted of developing a participant specific error window based on a 95% confidence interval around their Post-Transformed Misplacement. They also assessed whether true pairwise swaps (a pair of Misassignments where the locations of two reconstructed items were interchanged) were indeed the predominant error in hippocampal amnesia, including a reanalysis of the Watson et al. (2013) data. The data from Horecka et al. (2018) demonstrated three key findings: 1) hippocampal lesions resulted in significantly fewer Accurate Assignments and significantly more Misassignments, 2) overall Location Memory was not impacted by hippocampal lesions, who used just as many studied locations as controls, and 3) “true” Swap errors were uncommon and their propensity did not differ between amnestic patients and their matched controls. The findings suggest that spatial relational memory impairments in amnesia are perhaps even more severe than previously thought. The implication of a “swap” error is that participants knew the locations of two items and simply flipped them, which implies some level of relational memory. However, the Horecka et al. (2018) data make it clear that Misassignments are not systematic. Despite being able to remember and use a similar number of studied locations, hippocampal patients saw a complete loss of the ability to bind objects to specific locations.

The field of SRT has remained divided in recent years on the usage of the Watson et al. (2013) Axis Swap measures and the Horecka et al. (2018) Assignment-based measures. In truth, both measures are likely capturing much of the same hippocampally-mediated function, just at different levels of specificity. Even if the interpretation of Axis Swaps is perhaps misleading, their utility as a measure is undeniable, having been tied to neuroimaging, eye-tracking, and aging effects (Watson et al., 2013; Schwarb et al., 2016; Lucas et al., 2019, 2023). Whether the different kinds of “swaps” are tied to different kinds of processing is an interesting question which hopefully will be explored in future work. Moreover, it is possible that other types of neural dysfunction may increase swap errors, but to our knowledge this has not been directly studied in the context of spatial reconstruction. While in the following section we will survey the utility of SRTs in domains beyond hippocampal amnesia, it remains critical that the field moves toward consistency in its analyses. It is our recommendation that Assignment-based measures be the standard, as they more directly account for the underlying nature of spatial reconstruction performance. Assignment-based measures include the multiple levels of Misplacement assessment, allowing for complementary results. If Axis Swaps continue to be used, their operationalized definition needs to be more clearly outlined in those studies, and a deeper analysis of their underlying nature may be necessary.

## 4 Beyond amnesia: SRT as a measure of hippocampal function

Regardless of the preferred measure, SRTs have clearly established themselves as being able to index spatial memory impairments in hippocampal amnesia. Thus, much of the field shifted into investigating whether SRTs are sensitive to more subtle changes in spatial memory. Subsequent work extended the SRT across a broad range of populations including TBI, older adults, and children, while also incorporating complementary methods such as neuroimaging and eye-tracking. In many ways, it is here that SRTs really shine as a powerful tool in memory research, demonstrating a tremendous sensitivity to 1) alterations in spatial memory performance across multiple populations, 2) the strategic deployment of attention as indexed by eye-tracking, and 3) hippocampal integrity as measured via neuroimaging.

### 4.1 SRT in development, aging, and brain injury

Building on the findings in hippocampal amnestic patients, SRTs have been extended to multiple populations as an assessment of hippocampal function (though see section 5.3 for a caveat on such assumptions). The SRT has since been employed to assess the development of relational memory in children (Hassevoort et al., 2018), alterations in relational memory performance in aging (Clark et al., 2017) and Mild Cognitive Impairment (Tinney et al., 2023), the impact of Traumatic Brain Injury (TBI) on spatial memory (Rigon et al., 2020) and the relationship between fitness and nutrition with spatial memory performance (Hassevoort et al., 2017; Schwarb et al., 2017; Cannavale et al., 2019). Across these populations, SRTs have provided a sensitive framework for characterizing relational memory, with several consistent patterns emerging. For example, individuals with TBI display a similar pattern of deficits as individuals with hippocampal lesions, showing deficits in object-location binding measures but relative preservation of the general spatial configuration memory (Rigon et al., 2020). Older adults also show impairments in spatial reconstruction, including increased misplacement and relational errors (Clark et al., 2017), which are often interpreted as reflecting age-related declines in hippocampal-dependent binding processes. On the other end of the aging spectrum, developmental work suggests that children can represent the general spatial layout of an array but continue to show prolonged development in their ability to bind specific items to specific locations (Hassevoort et al., 2020). Critically, in both aging and early development, SRT performance has been shown to be distinct from mnemonic discrimination ability (Clark et al., 2017; Hassevoort et al., 2020). From these findings, SRTs appear to capture a hippocampally-dependent form of spatial-relational memory that is distinct from mnemonic discrimination, often linked to pattern separation (Yassa et al., 2011). That said, whether pattern separation plays a key role in spatial reconstruction may depend on the nature of the task parameters, something that has not been directly assessed as of yet. Across populations, spatial reconstruction tasks offer a powerful framework for isolating the representational structure of memory. Their ability to dissociate relational binding from coarse spatial representations demonstrates that hippocampal contributions to memory are best understood not as supporting spatial memory broadly, but as supporting high-resolution binding of items within spatial contexts.

### 4.2 SRT and eye-tracking: memory-guided strategic search

SRTs have increasing utility when coupled with other techniques, such as eye-tracking and neuroimaging, to examine the mechanisms that support spatial memory performance. Eye-tracking studies of spatial reconstruction have been leveraged to examine memory-guided information sampling during study, showing that particular patterns of visual search are closely related to subsequent reconstruction accuracy (Lucas et al., 2019, 2023). More specifically, studies have shown that more strategic and relationally focused viewing patterns lead to fewer spatial memory errors, whereas more entropic or disorganized viewing is associated with poorer performance in younger adults (Lucas et al., 2019) and older adults (Lucas et al., 2023). Importantly, individuals with hippocampal damage, in addition to showing increased spatial memory errors, also showed elevated entropy during study, despite making a similar number of fixations and transitions as healthy controls. These findings suggest hippocampal damage impairs not just performance, but also increases the entropy of study-time viewing, indicating that the hippocampus plays a key role in online information sampling and the formation of spatial relationships. Lucas et al. (2023) extended this work by showing the influence of the timing of viewing behavior during encoding for spatial memory formation. The findings from the study suggest that successful encoding may initially require focused processing of individual items before broader relational sampling across the display becomes advantageous. Additionally, early viewing behavior was related to later viewing patterns, indicating that encoding is a dynamic and interactive process. Together, the studies utilizing eye-tracking and SRTs suggest that the hippocampus supports not only the formation of relational representations, but also the dynamic coordination of information sampling across time during encoding. The role of visual attention in spatial reconstruction is still a relatively new avenue of research. Like other SRT research avenues, there is little data on whether other regions/processes may also influence strategic viewing behavior. Further, to date no eye-tracking study has investigated the role of attention during actual reconstruction, likely in part due to difficulties tracking attention while participants are moving objects. Nevertheless, combining eye-tracking with SRT provides a promising and rich set of data for future research.

### 4.3 SRTs and neuroimaging: key findings (and challenges)

In all of the work surveyed in the previous sections, authors have assumed SRT effects are tied to hippocampal changes based on the groundwork laid by studies in hippocampal amnesia. However, neuroimaging is necessary to confirm such inferences in non-amnestic populations. For the most part, emerging neuroimaging work has supported this interpretation, solidifying the utility of the SRT in assessing hippocampally-mediated spatial memory. Specifically, SRT performance has been strongly tied to hippocampal integrity, as measured by neuroimaging studies employing Magnetic Resonance Elastography (MRE) (Schwarb et al., 2016, 2017; Johnson et al., 2018; Daugherty et al., 2020; Hiscox et al., 2020; Delgorio et al., 2022). SRT performance is not only tied to hippocampal integrity in older adult populations (Hiscox et al., 2020) but even in a relatively homogenous young adult population (Schwarb et al., 2016). Further, Johnson et al. (2018) demonstrated that the SRT is uniquely tied to hippocampal and not orbitofrontal integrity, while fluid intelligence showed the reverse pattern, revealing a double dissociation between structure and function. Recent work has attempted to localize SRT performance with specific subfields of the hippocampus, though these findings have been somewhat inconsistent. Daugherty et al. (2020) found that SRT performance was tied to CA3-Dentate Gyrus integrity (as measured by MRE damping ratio) in young adults, while Delgorio et al. (2022) showed that SRT performance was tied to CA1-CA2 integrity (as measured by MRE stiffness). Given these data are from separate populations, it is possible they are not incompatible, and it is likely that multiple hippocampal subfields contribute to SRT performance. Regardless, the mounting neuroimaging data make it clear that SRT is an excellent index of hippocampal integrity across multiplepopulations.

However, despite its success with structural neuroimaging techniques, there is an overall lack of functional neuroimaging findings with SRT, which may contribute to the limited findings of brain regions associated with SRT outside of the hippocampus. To our knowledge, no study has employed a traditional SRT during a functional scan. This may in part stem from complexities of performing an SRT in the scanner: SRTs generally allow for untimed reconstruction and require a mouse to drag and drop items into their studied locations, meaning participants can reconstruct items in any order, move items multiple times, and take as much time as needed to complete the array. These features make the task difficult to implement in fMRI, where trial timing, movement, and response structure must be more tightly controlled. Further, control over study-time processing is limited; participants can sample the items in a variety of orders, thus the best way to tie such functional imaging data to subsequent reconstruction performance is unclear. The most straightforward solution, without altering the task itself, would be a block design, where trials are grouped based on overall spatial memory success (e.g., number of Accurate Assignments or overall misplacement). However, potential task adaptations may allow for event-related effects to be examined. For example, cueing participants to select the item that belongs in a specific location or using a grid format in which participants choose the item corresponding to each grid position may allow for investigation of the neural correlates of object-location binding success at an item-location level. However, although these adaptations may improve scanner compatibility, they also constrain the open-ended reconstructive processing that is central to SRTs.

Perhaps the closest example of functional neuroimaging using something like spatial reconstruction comes from Cooper and Ritchey (2019). In this study, participants studied object-locations embedded in scenes, one at a time. During retrieval participants were instructed to reconstruct each object's color and location by moving around a 360-degree color panorama and scene. Critically, this study showed that the hippocampus was not only involved in both encoding and retrieval of object-location information, but that it also interacted with posterior-medial and anterior-temporal networks differentially. Moreover, these changes in connectivity patterns were unique for color vs. spatial reconstruction performance. The findings highlight two key points: 1) the hippocampus of course does not work alone in support of SRT performance, and 2) without additional neuroimaging studies, the true nature of the various brain regions and processes that underlie spatial memory remains unknown. This gap in knowledge about the other regions and networks that support spatial reconstruction is a critical one to fill. Most, if not all, studies examining group differences in SRT performance claim such differences stem from hippocampal alterations. We caution that such assertions remain unproven without future functional neuroimaging studies. While it is true that SRT performance has been dissociated from orbitofrontal integrity (Johnson et al., 2018) and from frontal lesions (Smith and Milner, 1984), such findings do not imply other regions or networks do not contribute uniquely to spatial memory performance, nor does it rule out that alterations in other regions/networks may underlie differences in other groups beyond amnestic patients.

Considering a broader network implicated in SRT performance may help clarify how individuals encode, maintain, and reconstruct both item-specific information and the spatial relationships among items. As noted, the parietal cortex, particularly the posterior parietal cortex (PPC), is part of the dorsal “where” pathway and has been implicated in spatial processing (Sack, 2009). More specifically, the PPC has been linked to visuospatial processing (Sestieri et al., 2017), the planning of visually guided actions such as orienting gaze and reaching toward objects (Gottlieb, 2007), and the allocation of spatial attention (Capotosto et al., 2012). Furthermore, Sack (2009) suggests that overlapping PPC regions are engaged during both perceptual visuospatial tasks and spatial imagery, raising the possibility that the PPC may support not only the initial perception of spatial layouts but also the mental reconstruction of those layouts during retrieval. Interestingly, the parietal cortex is also critical for episodic memory (Wagner et al., 2005; Cabeza et al., 2008; Gilmore et al., 2015), though its causal role in memory retrieval is still somewhat debated (Rugg, 2022). Yet, the unique role of the parietal cortex in SRT performance is unknown. Similarly, the parahippocampal cortex, including the parahippocampal place area (PPA), is well known to be involved in the global processing of spatial layout and relationships between objects, rather than the remembering individual objects in an array or scene (Epstein and Kanwisher, 1998; Peer and Epstein, 2025). The PPA, along with other visual regions (Bonner and Epstein, 2018; Park et al., 2011) are critical for scene perception (Epstein and Baker, 2019), and may underlie our ability to encode multiple elements within a shared spatial context and to remember how these elements are organized in relation to one another, as tested in an SRT. Further, SRTs require participants to physically move objects (either on a tabletop or on a computer screen), meaning performance may be tied to motor planning functions of the PPC and motor regions such as the supplementary motor area (Ren et al., 2019), the premotor cortex (Schiavetto et al., 2002), and the primary motor cortex (Ren et al., 2019). Thus, future SRT studies must expand their scope to understand the regions that contribute to SRT performance as well as to disentangle the causal role of these regions and their processes in spatial reconstruction.

### 4.4 Reconstruction and navigation: different routes to the same processes?

While the focus of this review has been on SRTs in which participants reconstruct arrays of objects, studies of spatial navigation are also worth discussing. Navigation, whether virtual or real-world, engages many of the same cognitive demands and spatial processes as SRTs. Navigation depends on both allocentric and egocentric representations of space. Allocentric information may include object-to-object or landmark-to-landmark relationships, whereas egocentric information represents the environment relative to the self (Ekstrom et al., 2014). Interestingly, multiple navigation studies have included allocentric reconstruction tests post-navigation, many of which use similar methods to SRTs. Recent work using virtual navigation and Augmented Reality (AR) approaches have assessed precise object-location memory (Munoz-Montoya et al., 2019; Llana et al., 2024) as well as the geometrical relationship between objects and virtual rooms in which the objects' locations were reconstructed across different precision scales (Evensmoen et al., 2015). Navigation studies have also investigated the potential hierarchical organization of space (e.g., contextual/room vs. specific spatial location information), demonstrating that spatial information is organized hierarchically and information must be actively integrated across distinct subspaces (Peer and Epstein, 2025). To date, traditional SRTs have only scratched the surface of contextual influences on reconstruction performance.

As noted, navigation studies often consider both egocentric and allocentric representations. Conversely, SRTs have primarily emphasized only the allocentric components of spatial representation, likely because participants are asked to reconstruct the relationships among items in a view-invariant fashion. Accordingly, egocentric information may be less apparent in traditional SRT paradigms because it is rarely manipulated directly; however, this does not mean that egocentric processing is entirely uninvolved in SRT performance. Indeed, Srokova et al. (2026) suggest that spatial memory for object locations and navigation affordances may be closely intertwined. In this study, participants viewed arrays of objects on a table in a virtual environment and then were tested on whether the array was the same or different after a rotation. Notably, this rotation occurred either because the table itself rotated or because the participant walked around the table, with results showing better performance following self-movement. While this study did not include reconstruction, similar designs could be applied to SRT research to examine how changes in viewpoint and self-movement influence memory for object-to-object relationships within an array.

The hippocampus is also critically involved in spatial navigation and the formation of spatial memories, particularly when spatial relationships must be encoded and later reconstructed. Extending this work, Stokes et al. (2015) showed that hippocampal subfields make distinct contributions to the differentiation and integration of spatial context across subspaces. Similarly, Evensmoen et al. (2015) used a virtual navigation task during fMRI and later asked participants to reconstruct the allocentric locations of studied objects, a procedure conceptually similar to the SRT. Spatial accuracy was assessed using Positional Granularity, a measure of reconstruction precision that is independent of object identity. Results showed that the posterior hippocampus is associated with fine grained spatial representations, while the intermediate hippocampus was associated with medium grained representations, and the anterior hippocampus was associated with general object locations. The data suggest that spatial memory specificity varies along the long-axis of the hippocampus, something that has not been assessed in traditional SRTs. Additional research has also shown that remembering the layout of objects relative to the geometry of the room and its boundaries is tied to the anterior hippocampus (Evensmoen et al., 2021). Together, these findings suggest that successful spatial memory depends on the ability to distinguish similar spatial configurations while also integrating item, context, and reference-frame information into a coherent representation. These findings may also be relevant to SRTs, which have historically emphasized the relational structure among items in an array while giving less attention to the potential role of egocentric reference frames.

The navigation literature has also done a better job than the SRT literature at investigating contributions from regions outside of the hippocampus. For example, recent work has shown that that information about object position is linked to the posterior-medial entorhinal cortex and medial perirhinal cortex, while object identity is more associated with the anterior-lateral entorhinal cortex and lateral perirhinal cortex (Evensmoen et al., 2021). Further, the PPA has been implicated in navigation (Epstein and Baker, 2019), and recent data suggest that allocentric memory for object-locations after navigation may be supported by multiple MTL nodes (Evensmoen et al., 2021). This work has suggested that the MTL may not be organized hierarchically beneath the hippocampus, and the role of various MTL nodes may depend on the level or type of representation being tested. Beyond the MTL, research on allocentric spatial representations after navigation has implicated parietal, frontal, and visually responsive regions beyond the hippocampus (Ekstrom et al., 2014). Further, the microstructural integrity of white matter tracts, including the inferior longitudinal fasciculus, middle longitudinal fasciculus, and fornix, has been associated with more accurate representations of object-object relationships (Evensmoen et al., 2025) suggesting that successful spatial reconstruction may depend not only on regional hippocampal function but also on broader structural connectivity supporting relational spatial memory. How these regions and processes interact in service of SRT performance is an interesting question and worth future investigation.

As noted, there are clear differences in SRTs and spatial navigation tasks. To our knowledge however, no study has assessed whether and how performance on a standard SRT compares to navigation and subsequent allocentric map reconstruction. Recent work has suggested that eye movements during navigation may be critical in structuring navigation and subsequent memory (Zubair et al., 2026). Such findings parallel the emerging literature on the role of eye movements in SRTs (Lucas et al., 2019, 2023), and offer an exciting avenue for future research. As SRTs continue to evolve, incorporating elements from the navigation literature, like context and hierarchical organization, will be critical for understanding how the hippocampus similarly or distinctly supports allocentric reconstruction and spatial navigation. Conversely, SRTs' analysis pipeline may prove useful in assessing navigation-based spatial memory; while several navigation studies test subsequent reconstruction, measures like Accurate Assignments vs. Misassignments are rarely if ever used. Including such measures may further clarify the hierarchical organization of spatial information in the MTL, as well as the critical role of additional cortical regions.

### 4.5 Lingering inconsistencies and limitations in SRT

While the field of spatial reconstruction has begun to expand into different populations and techniques, such expansion has brought with it a great deal of variability in the exact nature of each implementation of SRT (See Table 2 for an outline of the various parameters that differ across tasks). To start, as previously noted, SRTs have been employed both with physical, real-world objects (e.g., Smith and Milner, 1981, 1984) as well as via computer-based reconstruction (e.g., Horecka et al., 2018; Schwarb et al., 2024), and could potentially be applied with advancements in Virtual and Augmented reality approaches (Munoz-Montoya et al., 2019; Zhou and Mou, 2019; Llana et al., 2024; Hill and Ekstrom, 2026). To our knowledge, no study has attempted to assess whether performance is correlated between the different apparatuses, let alone how such differences may impact different groups. Even when the modality is held constant, namely computer-based SRTs, the stimulus types used across spatial reconstruction studies have varied widely, including images of real-world objects that either share or do not share semantic category structure, abstract shapes, and novel “creatures” (Schwarb et al., 2024; Cannavale et al., 2019; Horecka et al., 2018; Watson et al., 2013). Additionally, studies have varied in the number of studied stimuli, the allocated study time, and the time interval used for the delay (Table 2). Differences in study time per item in the array and the delay interval may contribute to variability in spatial reconstruction performance across studies. Beyond differences in timing, SRT performance variability likely stems from multiple additional factors, some of which may simply be practical. For example, in children's SRT studies, novel “creature” stimuli are often used (Hassevoort et al., 2017, 2018, 2020), likely because more attention-drawing stimuli may be necessary to encourage engagement in this population. Similarly, for certain groups, there may be an upper limit to how many items can be studied in a fixed amount of time (Crane and Milner, 2005; Jeneson et al., 2010; Clark et al., 2017).

**Table 2:** Task design characteristics across computerized spatial reconstruction studies.

Stimulus type	Number of stimuli	Study time	Delay time	References
Real-world	5	16 s	5 s	Horecka et al., 2018
	6	16 s	4 s	Lucas et al., 2019 (Experiment 2)
	8	24 s	4 s	Schwarb et al., 2024 (Experiment 2)
Abstract shapes	3–5	Up to a minute	5 s	Clark et al., 2017
	5	20 s	4 s	Schwarb et al., 2016, 2017; Daugherty et al., 2020; Delgorio et al., 2022; Rigon et al., 2020; Tinney et al., 2023; Lucas et al., 2023 (Experiment 1)
	6	16 s	4 s	Lucas et al., 2019 (Experiment 1); Schwarb et al., 2024 (Experiment 1); Lucas et al., 2023 (Experiment 2)
	6	20 s	4 s	Johnson et al., 2018
Creatures or Animal-morphs	5	20 s	4 s	Hassevoort et al., 2017, 2018, 2020
	6	16 s	4 s	Schwarb et al., 2024 (Experiment 3)

Studies are organized by stimulus type and task parameters, including the number of stimuli presented in each array, study duration, and delay interval. All studies in the table used a mouse-based reconstruction procedure and allowed participants unlimited time to recreate the studied item array.

The general ambivalence to the nature of the stimuli themselves likely stems from relational memory theory, which posits that the hippocampus supports the binding of relational information regardless of stimulus domain (Konkel et al., 2008). Despite this claim, emerging evidence suggests that the nature of the stimuli can systematically influence performance. For example, SRT paradigms that incorporate semantic categories demonstrate increased relational binding errors within categories relative to between categories (Schwarb et al., 2024), indicating that pre-existing semantic structure can shape relational memory performance. Further, Schwarb et al. (2024) had three experiments, each with different types of stimuli (abstract squiggles, real-world objects, and Cat-Dog morphed stimuli). While differences were never assessed across experiments, the number of stimuli included in each experiment shifted: six abstract stimuli were used in Experiment 1, but eight real-world stimuli were used in Experiment 2. Interestingly, despite having more stimuli in Experiment 2, participants made, at least numerically, fewer relational errors compared to the abstract stimuli condition of Experiment 1 (though this difference was never assessed). Additionally, the question of how set size and study-test delay interact with SRT performance remains unresolved, let alone how such factors interact with different types of stimuli. Thus, while it may be a “feature” of SRTs that many different stimuli can be slotted into the arrays, such variability leaves many open questions. Without a better understanding of the interactions between these task dimensions and relational memory, there will remain substantial unexplained variability in how performance is realized across studies. Future studies should aim to systematically disentangle the role of stimulus type, array load, delay, and response format to better understand the nature of spatial memory and its interactions with these dimensions.

Although SRT tasks are sensitive measures of spatial reconstruction across populations, are easily modifiable, and provide rich data, they are not without limitations. As noted, because reconstruction requires interactive placement of objects, these tasks are difficult to administer during functional MRI studies, where motor movements are constrained and locking of item-level performance to particular time points may be difficult. Second, and relatedly, spatial reconstruction performance can be affected by motoric impairments, such as reduced fine-motor control or slowed movement, which may lead to increased placement errors unrelated to memory. This concern is especially relevant in clinical populations such as TBI, stroke, Parkinson's Disease, and even healthy aging (Mazzoni et al., 2012; Lodha et al., 2019; Corrigan et al., 2023; Seidler et al., 2010). Further, visual acuity can influence performance, since participants must locate objects and precisely interpret spatial positions on a screen or board. Visual deficits, whether due to aging, neurological damage, or individual differences may affect encoding of the objects in the array. Such visual deficits would then in turn affect spatial memory performance without reflecting underlying mechanisms of memory. The frequency of SRTs including measures of visual acuity and oculomotor control is relatively low; we suggest including visual acuity measures should become more standard. Lastly, it is possible, and necessary to mention, that SRT is not *the* measure of hippocampal function, but rather *a* measure. As briefly noted, SRT dissociates from other tests of hippocampal memory (e.g., mnemonic discrimination, spatial navigation), suggesting that it does not fully encompass relational memory processing. Even as a measure of hippocampal function, it remains unknown how other brain regions and networks uniquely contribute to SRT performance. While SRT has proven to be a powerful tool for understanding hippocampal spatial memory, these limitations highlight the need for careful consideration of task design across modalities and populations for spatial reconstruction tasks.

## 5 Discussion and future directions

While some variability in SRT administration is unavoidable, and likely scientifically interesting, we call for at least some improvement in standardization. We recommend that future studies move toward a standardized approach for analytical strategies, following the analytical steps outlined in Horecka et al. (2018). Raw misplacement remains important because it provides an unfiltered overall index of reconstruction accuracy; however, it cannot determine why errors occur. Remapped and Post-Transformed Misplacement measures provide additional information by separating identity-related errors and global transformation errors. Moreover, those processing steps allow for Assignment-based measures, including Accurate Assignments and Misassignments. These measures are thought to reflect hippocampal object-location binding, though they are likely an indirect reflection of this process and are also affected by other variables. Regardless, the strength of Assignment-based measures is that they assess accuracy, providing a more targeted estimate of binding accuracy rather than solely measuring error. Further, using these measures may clarify whether group differences reflect failures of binding, location memory, or broader reconstruction precision. Further, while Axis Swap measures are popular, if they continue to be used, they should be operationalized clearly and interpreted cautiously, because inverted vectors do not necessarily indicate true object-location swaps.

Future work should also expand the use of multimodal approaches. Eye-tracking has already shown promise in uncovering the nature of strategic encoding, but can further clarify how visual sampling patterns during encoding and retrieval support reconstruction, and how these effects interact across different groups or task dimensions. Additional functional neuroimaging studies will be critical in identifying and characterizing the broader neural networks involved in spatial reconstruction beyond the hippocampus, both during encoding and retrieval. This is especially important because SRT performance likely reflects coordinated interactions among hippocampal, prefrontal, parietal, and medial temporal regions. It is imperative that the cognitive processes and neural correlates of the various SRT measures are more clearly outlined. The focus on hippocampal-dependence will always be a compelling question, but understanding how frontal, parietal, MTL, and other regions map onto SRT performance, and dissociate across measures, is a critical next step. Further, SRTs may have value beyond basic spatial memory research. Because they are sensitive to subtle disruptions in relational memory, SRTs may be useful for detecting early cognitive changes in clinical groups and for tracking memory changes across development, aging, and intervention studies. Given the relative ease of administering SRTs, development and norming of clinical assessment variants of SRTs may prove useful in uncovering distinct, or shared, deficits in spatial memory within and across clinical populations. Such work could lead to not only better diagnostic assessments, but opportunities to develop reconstruction-based training and/orinterventions.

Lastly, reconstruction-based approaches may prove fruitful in understanding the reconstructive nature of memory beyond space, such as in temporal order memory, object-feature binding, or even semantic relations. Overall, SRTs are not simply measures of spatial accuracy; they offer a window into how memories are organized, bound, and reconstructed (Jenkins and Ranganath, 2010). Many temporal memory tasks, such as those involving event segmentation or temporal order memory, require individuals to reconstruct when events occurred (Zacks and Swallow, 2007; DuBrow and Davachi, 2014). Both spatial and temporal memory require hippocampally-mediated integration between the relationships among elements, and differentiation between distinct (Bellmund et al., 2022; Evensmoen et al., 2025). Further, recent work from our group demonstrated that using SRT misplacement and Assignment measures in a simple temporal order task may reveal memory deficits in TBI beyond standardized (Dulas et al., 2022), suggesting such measures have utility beyond spatial processing. By improving the consistency in design and analysis, SRT measures may prove useful in fully understanding the specificity, resolution, and reconstructive nature of memory across domains.

### 5.1 Conclusion

So where are we at with Spatial Reconstruction? Spatial Reconstruction Tasks have emerged as a powerful tool for assessing relational memory. They are 1) relatively simple to conduct, yet rich in their data output, 2) adaptable across multiple apparatuses, stimulus domains, populations, and assessments, and 3) compatible with multiple converging techniques. Even with the large amount of variability across SRT studies, their ability to index hippocampal integrity and function is clear, with a particular focus on object-location binding. However, more work is needed to understand how other regions, processes, and non-mnemonic factors influence SRT performance. More standardization of analysis is a critical next step in the evolution of SRTs, that will allow better comparisons across tasks and research groups. Such standardization will unlock the full potential of SRT and its measures, allowing further connections with navigation and other memory functions. Further, applying SRT's misplacement, global change, and Assignment-based measures to other domains, such as temporal order memory, may allow a comprehensive understanding of the reconstructive nature of memory beyond just “space.” The research questions that SRT studies are bound to answer in the coming years will offer exciting insights into hippocampal function, spatial memory, and the reconstructive nature of our memories.

## Footnotes

1 One caveat that should be noted about the global transformation in Horecka et al., 2018: global transformation was only applied on trials if the transformation could be applied to ALL reconstructed points, with translation + scaling and rotation being allowed to fail independently. It is not reported how frequently global transformation “failed,” and it is also unknown whether transformations exist for subsets of an array. Further, while minor differences have been seen in the global transform metrics, these have been generally small and not deeply discussed in the literature (Horecka et al., 2018; Rigon et al., 2020).

2 In Horecka et al., 2018, these measures referred to as “Correct Item-Location and Identity-Location” (Assignments), “Correct Item-Location and Wrong Identity-Location” (Misassignments), and “Inaccurate Location” (Location Memory). Assignments have also been referred to as Object-Location Binding in multiple places (e.g., Cannavale et al., 2019; Hassevoort et al., 2018; Rigon et al., 2020). We elect to use Assignments, both for its brevity and because “Object-Location Binding” implies a process, rather than a measure.
